# Plant-Derived Lapachol Analogs as Selective Metalloprotease Inhibitors Against *Bothrops* Venom: A Review

**DOI:** 10.3390/ijms26093950

**Published:** 2025-04-22

**Authors:** Paulo A. Melo, Pâmella Dourila Nogueira-Souza, Mayara Amorim Romanelli, Marcelo A. Strauch, Marcelo de Oliveira Cesar, Marcos Monteiro-Machado, Fernando Chagas Patrão-Neto, Sabrina R. Gonsalez, Nilton Ghiotti Siqueira, Edgar Schaeffer, Paulo R. R. Costa, Alcides J. M. da Silva

**Affiliations:** 1 Laboratório de Farmacologia das Toxinas, Programa de Pós-Graduação em Farmacologia e Química Medicinal, Instituto de Ciências Biomédicas, Universidade Federal do Rio de Janeiro, Rio de Janeiro 21941-590, Brazil; pamdourila@gmail.com (P.D.N.-S.); mayararomanelli@yahoo.com.br (M.A.R.); m.ocesar@hotmail.com (M.d.O.C.); marcosmmachado@gmail.com (M.M.-M.); fcpatrao@gmail.com (F.C.P.-N.); 2 Departamento de Ciências Fisiológicas, Instituto de Ciências Biológicas e da Saúde, Universidade Federal Rural do Rio de Janeiro, Seropédica 23890-000, Brazil; strauchmarcelo@yahoo.com.br; 3 Instituto Vital Brazil, Niterói 24230-410, Brazil; 4 Faculdade de Medicina, Universidade Federal do Rio de Janeiro, Campus Macaé, Rio de Janeiro 21941-901, Brazil; srgonsalez@gmail.com; 5 Centro de Ciências da Saúde e do Desporto, Universidade Federal do Acre, Rio Branco 69920-900, Brazil; nilton_ghiotti@uol.com.br; 6 Instituto de Pesquisas de Produtos Naturais Walter Mors, Centro de Ciências da Saúde, Universidade Federal do Rio de Janeiro, Rio de Janeiro 21941-590, Brazil; edgar.ippn@gmail.com (E.S.); prrcosta2011@gmail.com (P.R.R.C.); alcides@ippn.ufrj.br (A.J.M.d.S.)

**Keywords:** snake venom, *Bothrops atrox*, metalloproteinases, tissue hemorrhage, plant compounds, lapachol analogs, synthetic inhibitors

## Abstract

Plant compounds that inhibit snake venom activities are relevant and can provide active molecules to counteract snake venom effects. Numerous studies on snake viperid venoms found that metalloproteinases play a significant role in the pathophysiology of hemorrhage that occurs on envenomation. Preclinical studies using vitro and in vivo protocols investigated natural compounds and viperid snake venoms, evaluating the enzymatic, procoagulant, hemorrhagic, edematogenic, myotoxic, and lethal activities. Many studies focused on *Bothrops* venoms and ascribed that angiorrhexis and hemorrhage resulted from the metalloproteinase action on collagen in the basal lamina. This effect resulted in a combined action with phospholipase A2 and hyaluronidase, inducing hemorrhage, edema, and necrosis. Due to the lack of efficient antivenoms in remote areas, traditional native plant treatments remain common, especially in the Amazon. Our group studied plant extracts, isolated compounds, and lapachol synthetic derivative analogs with selective inhibition for *Bothrops* venom proteolytic and hemorrhagic activity and devoid of phospholipase activity. We highlight those new synthetic naphthoquinones which inhibit snake venom metalloproteinases and that are devoid of other venom enzyme inhibition. This review shows the potential use of snake venom effects, mainly *Bothrops* venom metalloproteinase activity, as a tool to identify and develop new active molecules against hemorrhagic effects.

## 1. Introduction

Snakebites occur worldwide, causing disabling injuries and death, with a profound social impact in rural settings, most often in Africa, Asia, and Latin America [1,2,3,4]. Snakebite is classified as a neglected tropical disease that most global health authorities ignore, and the envenoming can impose a significant economic burden on a poor population [1,2,3,4,5,6,7,8,9]. Many victims do not attend health centers or hospitals and rely on traditional treatments, which makes the present data inaccurate, as stated by the World Health Organization (WHO) [10,11]. The WHO reports that around 4.5–5.4 million people are bitten by snakes annually, and an estimated of 1.8 to 2.7 million people develop clinical illness from snakebites annually, with 81,000 to 138,000 deaths attributed to related complications [11]. Viperid venoms are a cocktail of pharmacologically active toxins or proteins that have been under investigation since the earliest biochemical studies on snake venoms [12,13,14,15,16]. A substantial number of these proteins were isolated and structurally, biologically, and biochemically well characterized. Some were used as tools in fundamental physiology and pharmacology discoveries [7,8,14,17,18]. Approximately 90–95% of the dry weight of snake venom corresponds to proteins and peptides that act as toxins, and it may or may not have enzymatic action. This composition can be made up of phospholipase A2 (PLA2), metalloproteinases (SVMPs), serine proteases (SVSPs), L-amino oxidases (LAAOs), phosphodiesterases (PDEs), hyaluronidases (HAases), acetylcolinesterases (AchEs), nucleases, three-finger toxins, disintegrins, cysteine-rich secretory proteins, and C-type lectins [13,19]. Among these complex components are specific protein families that exhibit pleiotropic enzymatic properties, such as PLA2, SVMP, SVSP, hyaluronidase, and LAAO [8,12,13,15,17,18,20,21,22,23]. Snake venoms are integrated multicomponent systems that may act synergistically. Those protein complexes can increase or decrease the pathophysiological effects of envenomation in victims [16,22,24]. The main local effects of viperid snake venom, when inoculated, are hemorrhage, edema, and local tissue necrosis [25,26,27,28,29,30]. Many published data describe that, among the Latin America viperid snakes, *Bothrops* species are responsible for most of the envenomations, leading to significant morbidity and socioeconomic burden in developing countries [1,10]. Most of the snakebite accidents in the Brazilian Amazon result from *Bothrops atrox* envenomation [10,30,31,32,33,34,35,36].

Snakebite treatment and first aid in these accidents are challenged by multiple factors, which include the individual venom complexity and many other circumstances that can delay prompt medical support [4,13,36]. Attempting to treat a snakebite depends on the distance from health support and the distribution of the antivenom. That usually occurs in large areas far from health support, adding to the equation the occurrence of interspecies or genus variations, which complicate the snake identification [13,15,20,25,30,37]. In the South American continent, specifically in Amazon Brazilian borders, there are vast remote places without health support for the treatment of ophidian accidents with either specific or polyvalent antivenoms [3,9]. It is known that, in some situations, due to the lack of efficient and safe treatment in isolated areas, therapy with polyvalent antivenom is replaced by local folk medicine using herbs or medicinal plants [5,8,13,28,36,38,39,40,41,42]. While antivenoms remain the primary treatment, their availability is often limited in remote areas, reinforcing the need for alternative or complementary therapies [4,5]. Previous studies have proposed using potential medicinal plants to halt the effect of snake venoms. The traditional use of plants to treat snakebites is a common practice in the western Amazon in Brazil [5,41,43]. Other reports show that some plant extracts were able to inhibit snake venom-induced hemorrhage in vitro and in vivo. Many studies are being carried out to validate the traditional use of these species plants to treat snakebites [5,29,36,42,43,44,45,46,47,48,49,50]. Therefore, exploring plant-based therapeutic strategies represents a promising avenue for improving snakebite management, particularly in regions with restricted access to conventional treatments, especially in places lacking good health facilities or minimum resources, such as electricity and the structures required to provide access to the specific antivenom [10,42,43,45]. Previous investigations have shown that plants and their compounds can be effective antivenoms and provide the basis to develop new molecules [5,19,51,52,53]. In this context, preclinical research shows that Lapachol derivatives effectively inhibit SVMPs, decrease hemorrhage, and safeguard tissues, while leaving other venom enzymes, such as PLA2, unaffected [54]. The main objective of this review is to highlight that synthetic Lapachol analogs act as SVMP-specific inhibitors for *Bothrops* envenomation.

## 2. Materials and Methods

### Literature Review

A systematic literature search was conducted across electronic databases, including PubMed, ScienceDirect, and Web of Science, covering publications up to 2025. The search strategy incorporated the keywords “plants”, “snakebites”, “*Bothrops* envenomation”, “folk medicine”, “metalloproteinases”, “snake venom metalloproteinases”, “metalloproteinases inhibitors”, and “Lapachol”, which were combined using the Boolean operator “AND” to refine the results and to optimize the retrieval of relevant studies. The inclusion criteria encompassed studies evaluating tissue damage caused by *Bothrops* envenomation, focusing on the effects of metalloproteinases and their potential inhibitors, including Lapachol and its analogs. Studies lacking methodological details were excluded. Editorials, full texts, and peer-reviewed articles published in English were considered for further analysis. Most of the papers addressed the use of plants in folk medicine, particularly in remote areas. Additionally, some of the studies included previous reports from our research group demonstrating that isolated compounds from these plants exhibit antivenom activity. The results found can be observed on Table 1.

## 3. Previous Studies

As described previously, some of the studies reviewed in this work were prior studies reported by our group, demonstrating that natural plant metabolites can present antivenom effects, protecting the tissue from damage due to toxins in the snake venoms. Among the plants investigated are *Eclipta prostrata* (Asteraceae), *Combretum leprosum* (Combretaceae), *Humirianthera ampla* Miers (Icacinaceae), *Tabebuia impetiginosa* (Bignoniaceae), and *Aegiphila integrifolia* (Lamiaceae). These data showed that the crude plant extracts and isolated compounds from these plants antagonized the venom effects [29,38,39,46,47,54,55]. Our group has been particularly interested in searching for new alternative antagonists, derivatives from animals or plants, to treat or prevent the damage caused by snakebites [29,56,57,58,59,60]. In this context, we also investigated pharmacologically effective and planned synthetic active compounds, evaluated with many in vitro and in vivo studies [51,52,61]. Among the active compounds isolated and studied from a plant named *Eclipta prostrata* (EP) with antivenom activity, we have assessed wedelolactone (WEL), a natural coumestan, which is one of the most active compounds of the EP. WEL reproduced the EP crude extract effects and antagonized the muscle damage, the hemorrhagic effect, and the proteolytic and phospholipase activities of different Brazilian and North American snake venoms and their isolated toxins [5,55,59,62]. We advanced our research by synthesizing new coumestans with varying oxygenation patterns and by screening them for antivenom activity. These novel compounds replicated the effects of WEL, with one displaying antimyotoxic activity at an IC_50_ comparable to WEL [61]. Expanding this approach, we synthesized 8-methoxy coumestrol, a natural product typically found at very low yields when isolated from *Medicago sativa*, which exhibited similar activities to other coumestans [53,63].

Furthermore, we performed many in vivo tests against myotoxicity, edema, and hemorrhage induced by *Bothrops* venoms in mice. The protective effect of this coumestan against *Bothrops* venom was also studied by using isolated mouse skeletal muscle, rat heart Langendorff preparations, and phospholipase and proteolytic assays [53]. Additionally, we investigated of a pterocarpan named edunol, which was naturally found and isolated from *Harpalyce brasiliana*, a plant used in Brazil against snakebites [52]. This compound was obtained by synthesis and showed inhibitory properties against *Bothrops* snake venoms, such as antimyotoxic and antiproteolytic activities [52]. This investigation found that these proprieties could be improved by synthesizing a bioisoster by replacing the prenyl group with the benzyl group [52]. Two other listed plants, *Combretum leprosum* and *Humirianthera ampla*, were also investigated, and it was discovered that these plants and their isolated compounds, arjunolic acid and lupeol, inhibited the myotoxic, edematogenic, and anticoagulant activities of *Bothrops* venoms [29,46].

We continued our investigations with natural compounds by using *Tabebuia impetiginosa* (Bignoniaceae), a canopy tree with pink, yellow, white, and purple flowers found all over the South American continent that contains Lapachol [64,65], a naphthoquinone first isolated from *Tabebuia impetiginosa* in 1858 [54,66]. Gong and collaborators [67] demonstrated that *T. impetiginosa* and Lapachol derivatives exhibit antimalarial, antifungal, antibacterial, antiparasitic, and antitumor activities [67,68]. Our research focuses on Lapachol molecules as a starting point to obtain new bioactive naphthoquinone derivatives, which exhibit interesting pharmacological profiles [51,69,70]. Studies using different experimental protocols have demonstrated that synthetic naphthoquinones structurally related to Lapachol exhibit activity against tissue damage induced by *Bothrops* venoms [51,54]. The present contribution summarizes our recent investigations to understand how the tissue is targeted by *Bothrops* snake venom, which acts with combined toxins, thereby inducing different local reactions and damage. These data are relevant because the snakes from this genus are responsible for most snakebites reported in Brazilian Amazon borders [13,25,30,31,33,71].

**Figure 1:**
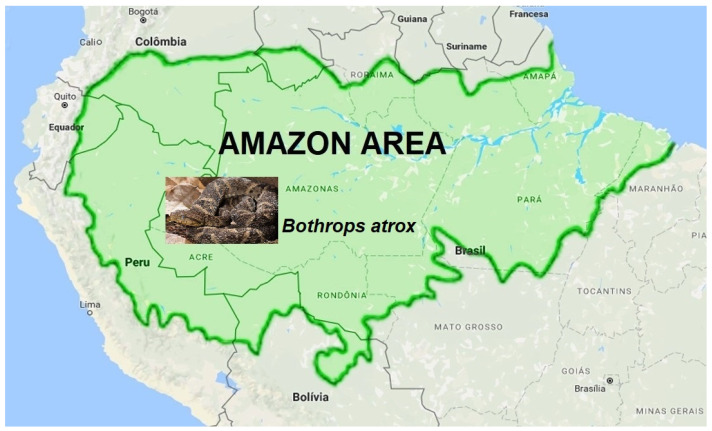
Geographic distribution of *Bothrops atrox* in South America and Brazil. Map exhibiting part of the South American continent and the north part of Brazil’s borders, showing the typical snake of this area, *B. atrox*.

Figure 1 denotes part of the South American continent, with the northern part of Brazil’s borders, showing the typical snake of this area, *B. atrox* [22,72]. Like the other snakes from the genus *Bothrops*, proteomic studies of the *B. atrox* venom present and a low amount of PLA2 and the predominant presence of metalloproteinases (SVMPs)—more than 30%—producing intense hemorrhage, bleeding disorders, and edema [10,17,22,31,34,72,73,74]. Furthermore, it is considered the most critical snake involved in human envenoming in the Amazon, which induces severe tissue hemorrhage [22,31,36,72].

## 4. Tissue Hemorrhage and Damage Induced by *Bothrops* Snake Venoms

*Bothrops* snake venoms contain toxins that target local tissues and blood hemostasis and are responsible for a broad range of clinical and biological syndromes, including local and systemic bleeding, blood incoagulability, thrombotic microangiopathy, and macrothrombosis [75]. Previous clinical and experimental observations described that local hemorrhage and systemic tissue damage are common consequences of envenomation due to *Bothrops* snakebites [2,3,9,20,28,32,76]. Those deleterious effects result from the action of *Bothrops* snake venoms, which contain complex components that are both nonenzymatic and active catalytic enzymes that act together, inflicting local and systemic effects [6,9,77]. Many investigations have assigned that, in the pathogenesis of these venoms, hemorrhage results from the direct damage of microvessels caused by hemorrhagic toxins, most of which are metalloproteinases [6,7,12,28,76]. These venom effects can result from the direct action of combined toxins producing endothelial cell damage, mainly by their proteolytic, phospholipase, and other activities [8]. Thus, it induces the activation of local mediators, thereby disrupting capillary vessels and producing angiorrhexis, edema, and inflammation [8,78,79]. They suggest that this late tissue damage and necrosis could be not only a consequence of the vascular damage but also the reduction in blood tissue supply and nutrition, which could induce necrosis [20,76,79]. Finally, there is the ability of snake venom metalloproteinases (SVMPs) to play biological roles in prey immobilization and digestion, hydrolyzing relevant protein substrates in the tissues [76,80,81]. The majority of SVMPs act by disrupting the vascular membrane by degrading the basement membrane and the extracellular matrix proteins, facilitating the local cell and fluid leakage after inducing local hemorrhage, thus forming local edema and late necrosis [6,7,8,74,79,80,82]. This damage, combined with the venom’s abilities to induce and increase the presence of very activate cytokines, promotes a massive inflammatory response, which may also be responsible for the capillary leakage of leukocytes to promote intense edema, thereby worsening the local inflammatory reaction [8,12,83,84].

**Figure 2:**
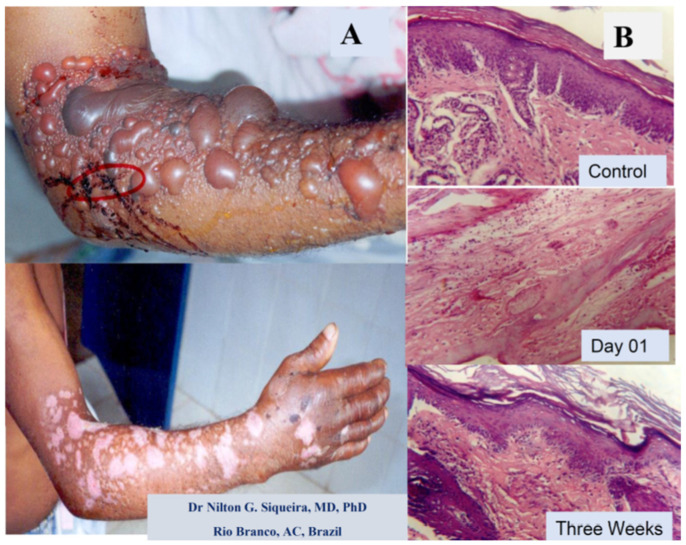
Effects of *Bothrops* snakebite on a human arm and a *Bothrops* snake venom injection in mice. (**A**) Day one and three weeks after cutaneous lesions induced by the *Bothrops* sp. snakebite in the adult male arm. Presence of blistering, local edema, skin hemorrhage, and necrosis. (**B**) Light microscopy section stained with hematoxylin and eosin of mouse skin before (control) and after *Bothrops* venom injection. The control, on day one after venom injection, with the presence of edema, inflammation, necrosis, and incomplete regeneration with skin disruption three weeks later. (Magnification 400×).

**Figure 3:**
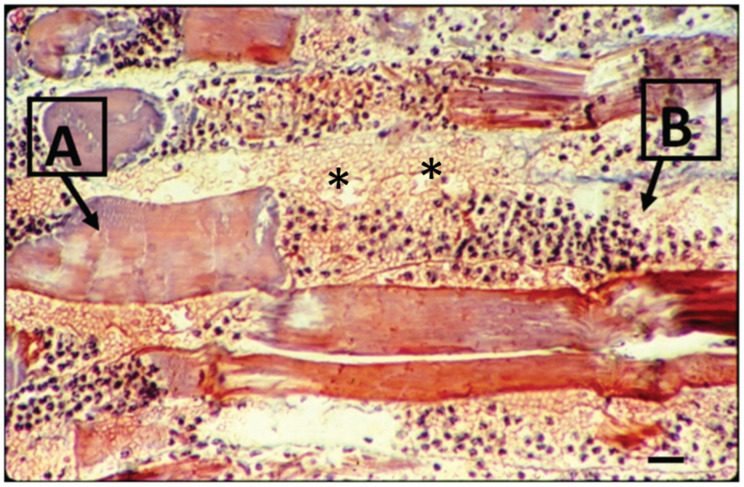
Light microscopy of mouse muscle 24 h after the perimuscular injection of *Bothrops* venom. The longitudinal section is stained with hematoxylin and eosin. Note the necrotic myofibers in different stages of degeneration, as shown in (A), and the intense hemorrhage and acute inflammatory infiltration on muscle fiber in (B). (*) indicates sarcolemma disruption patches (bar = 10 μm) (modified from Saturnino-Oliveira et al. [86]).

The clinical manifestations of tissue injury inflicted by snakebites in humans can be reproduced in experimental models using different protocols, as shown in Figure 2 and Figure 3, and many other reports [6,7,20,32]. These observations help to explain the complex tissue reactions, allowing investigators to understand the pathophysiology and to test different treatments so to enhance the effectiveness of antivenom therapy. Recent publications on snakebites have shown that tissue damage, such as hemorrhage, myonecrosis, and hemostasis disorders, results mainly from metalloproteinase and phospholipase activities with the involvement of immunological/inflammatory mediators [6,7,8,12,32,74,75].

An example of a human snakebite and experimental investigation are shown in Figure 2, with a picture of a damaged arm from a male Amazon worker from Acre state, Brazil, after a *Bothrops* snakebite. The patient arrived without any previous treatment, with a large skin injury, presenting blisters, edema, and late scars resulting from the necrotic and inflammatory reactions induced by the venom components (Figure 2A). The figure also shows the results of the experimental light microscopy of mouse skin before and after *Bothrops jararaca* venom injection (Figure 2B).

Figure 2B shows the tissue of mouse skin in the acute and late inflammatory reaction, partially reproducing the clinical critical situation. These images show that the damage induced by snakebite in humans could be reproduced in experimental conditions in animals, and allowed us to develop new therapies by testing new compounds from different sources with antivenom abilities, as well as developing many strategies to improve snakebite treatments [5,6,13,32,59,85].

While Figure 2A illustrates a real clinical situation observed after a snakebite (without any treatment), Figure 2B displays the experimental mouse skin effect induced by *Bothrops atrox* venom, showing the light micrography of the temporal impact (day one versus three weeks after the venom injection). These findings demonstrate a relationship between the clinical and experimental reproduction of tissue events induced by *Bothrops* venoms. Besides that, Figure 2 and Figure 3 qualitatively illustrate the main clinical manifestations—such as blister formation, edema, hemorrhage, and necrosis—with their corresponding histopathological alterations. Figure 3, showing a histological micrograph of skeletal muscle tissue stained with hematoxylin and eosin (HE), reveals structural damage consistent with the clinical observations. Region “A” highlights muscle fibers undergoing necrosis, characterized by morphological disorganization, while Region “B” displays a dense polymorphonuclear infiltrate, indicating an intense inflammatory process and associated hemorrhage. These findings reinforce the interplay between macroscopic and microscopic pathological events, providing a comprehensive understanding of the tissue alterations induced by *Bothrops* venom.

## 5. Snake Venom Metalloproteinases

The first description and discovery of zinc-dependent proteinase viperid snake venom was performed by Takahashi and Osaka [17,87]. After the discovery of bradykinin by Rocha e Silva et al. [88], the importance of the proteolytic activities of *Bothrops* snake venom compared to trypsin was magnified. It took many years until the enzymatic properties of the snake venom from this genus was investigated further [6,18,88,89,90]. Four decades ago, Doctor Fagja Mandelbaum and her colleagues at the Instituto Butantan in Brazil isolated bothropasin, a hemorrhagic toxin from *Bothrops jararaca* venom. It took several years until the determination of the structure of this snake venom metalloproteinase class was reported [91,92,93]. Many metalloproteinases (SVMPs) have been found and isolated from snake venoms, and their structure and mechanisms of action have been reported [18,76,89,90,94,95]. Other snake venom toxins and SVMPs were characterized and well documented in some reviews describing the properties of these enzymes, such as in a timeline of key events published by Fox and Serrano [96], as well as by others [6,17,18,76,77,85,89,94,97,98]. They describe that metalloproteinases are zinc-dependent enzymes present in different living forms, from bacteria to mammals, and play many physiological and pathophysiological roles in health and disease, such as proteolytic transmembrane proteases that modulate diverse cell functions and coordinate intercellular communication [98,99,100]. Previous reviews on hemorrhagic toxins raised the possibility that structural similarities were observed among these snake toxins, and that there were structural relationships between these toxins and the mammalian proteins [100]. Some metalloproteinases are localized at different compartments inside the cell, including the cytosol, cell organelles, and the nucleus. These intracellular proteases contribute to the pathogenesis of various diseases [101]. They have been traditionally grouped according to their extracellular matrix substrates, primary structure, or subcellular localization as gelatinases (MMP-2 and -9), collagenases (MMP-1, -8, and -13), stromelysins (MMP-3 and -10), matrilysins (MMP-7 and MMP-26), metalloelastases (MMP-12), and membrane-type MMPs (MMP-14–16 and MMP-23–25) [99]. Some examples of pathogenic actions include cardiovascular, renal disorders, inflammation, malignancy, and the uncontrolled or abnormal growth of cells or tissues in the body [12,101]. Metalloproteinases have long been described as matrix modulators and an important step enzyme in gene expression [102,103].

In the last decade, many publications have reported on the importance of metalloproteinases (MMPs), their relevance, and how they are thought to be essential in a diverse invasive process of angiogenesis and tumor metastasis [22,76,101,104,105,106,107]. Snake venoms are rich sources once they contain different amounts and mixtures of various proteins [6,7,96]. Many venoms of the Viperine and Crotaline families contain in their composition phospholipases, snake venom serine protease (SVSP), snake venom metalloproteinases (SVMPs), collagenases, and many different toxins or toxin isoforms of different molecular weights [18,22,85,97,105,108]. These metalloproteases or metal-dependent enzymes constitute a significant protein family in widely distributed viper venoms, and they are present in all of the viper species, playing a significant role in the pathophysiology of viperid envenomation and tissue damage [12,17,18,37,75,84,85,93,96]. Proteomic investigations showed that they represent more than 50% of the composition in Bothrops venoms, and they ascribed that the hemorrhagic activity plays a significant role in most SVMPs [89,94,96].

Snake venom metalloproteinases (SVMPs) are classified into various groups (P-I–IV) according to their composition [17,18,94,96]. However, the absence of proteomic evidence and the failure to detect a P-IV mRNA transcript led to reclassifying the P-IV class into the P-III class (P-IIId). It has been suggested that the P-IV class represents a post-translational modification of the established P-III structure, incorporating a lectin-like domain [98].

P-III SVMPs, comprising metalloproteinase, disintegrin-like, and cysteine-rich domains, exert more potent hemorrhagic activity than P-I SVMPs [6,7]. SVMPs also degrade various components of the basement membrane and hydrolyze endothelial cell membrane proteins, such as integrins and cadherins, which are involved in cell–matrix and cell–cell adhesion, acting as desintegrins [17,18,94,105]. Metalloproteinase family proteins constitute a significant class of membrane-anchored multi-domain proteinases that are responsible for the shedding of cell surface protein ectodomains, including the latent forms of growth factors, cytokines, receptors, and other molecules [12,96,97,98]. In addition, disintegrin-like and cysteine-rich domains interact with endothelial cell integrins, interfering with their adhesion to the extracellular matrix [96,97,98]. Studies have revealed that, over the years, most of these characterized metalloproteinases from snake venom (SVMPs) represent a diverse group of multi-domain proteins with several biological activities, such as the ability to induce hemorrhage, the proteolytic degradation of fibrinogen and fibrin, the induction of apoptosis, and the inhibition of platelet aggregation [96,97,98]. It is proposed that SVMP-induced hemorrhage acts in vivo by a two-step mechanism [6,7,8]. Initially, SVMPs degrade the basement membrane and adhesion proteins, thus weakening the capillary wall and perturbing the interactions between endothelial cells and the basement membrane [6,7]. The hemorrhage induced by SVMPs is a highly rapid event in vivo, with capillary endothelial cells showing drastic structural alterations within a few minutes [6,7].

In contrast, observations in cell culture conditions do not evidence such rapid endothelial cell damage [76,97]. Gutiérrez et al. [7] described the following: “Instead, the main effect is detachment and rounding of these cells; it is only after several hours of incubation that cells show evidence of apoptotic damage”. This apparent discrepancy between in vivo and in vitro observations could be explained by biophysical forces operating on microvessels in vivo. Taking into consideration that the transmural pressure acting on the weakened capillary wall can cause distention, endothelial cells become very thin until the integrity of the capillary wall is lost at some point, wherein extravasation occurs. In addition, endothelial cells become more susceptible to blood flow-dependent shear stress, which further contributes to capillary wall disruption [6,7,79]. These proteolytic enzymes from *Bothrops* snake venom are also zinc-dependent metalloproteinases, which are responsible for the hemorrhagic activity that is characteristic of this venom, which induce hemorrhage and tissue inflammatory response, edema, and tissue necrosis [6,7,31,76,109].

## 6. Potential Inhibitors of Snake Venom Metalloproteinases

Investigators worldwide search for natural and synthetic inhibitors that could replace or complement serotherapy on snakebite envenoming. Clare et al. [110] and others have described snakebites as a neglected and life-threatening tropical disease that causes more than one hundred thousand deaths each year, and there are many deficiencies associated with current biological antivenom therapies. Furthermore, they ascribed that many small-molecule drugs have demonstrated highly promising preclinical efficacy against snakebites, and the potential to administer such drugs orally in snakebite-affected communities offers an exciting new treatment strategy [110]. A promising find was the substance batimastat, which inhibited the hemorrhagic effect induced by *B. asper* venom in the lungs after intravenous injection, and delayed the time to death, as well the in vitro effects on hemostasis [111]. Additionally, the investigation of target protein binding and site prediction by using molecular docking analysis showed that batimastat complexed with a phytochemical compound, therefore inhibiting the venom metalloproteinase [112]. Compound **3a**, reported by Strauch et al. [54], inhibited venom metalloproteinase effects, such as hemorrhage, edema However, it was not tested on lethality effect or compared with other inhibitors [54]. As the chemical space explored for snakebite drugs is minimal, these authors also reinforced that drug discovery programs are urgently needed to broaden the snakebite drug portfolio and overcome the challenges of developing single-drug or combination drug therapies [110]. The diverse enabling strategies successfully used by drug discovery programs for other neglected tropical diseases provide promising avenues for delivering future snakebite therapeutics [4,110]. Furthermore, the WHO coordinates a global strategy to improve antivenom quality and develop small molecules to help neutralize different SVMPs [8,110,113]. They report that numerous promising inhibitors for metalloproteinases and PLA2 are being developed for the treatment of other human diseases, and they could have another purpose in medicine as a support for neglected diseases, as well as in snakebite treatments [110,114,115]. Although antivenom therapy is recognized to reduce the snakebite mortality rate and is the only safe therapeutic agent available [4], developing new small molecules that could reach the affected tissue and neutralize the snake venom metalloproteinases is necessary. These synthetic compounds could be evaluated and added to the arsenal of antivenom therapy in snakebite accidents [115,116].

Some authors have argued that metalloproteinase and its inhibitors are tools to develop and find new therapeutic agents. They can act as a target in different molecular approaches, such as in computer molecular models [117]. Preciado et al. [118] described the treatment of local tissue damage induced by venom metalloprotease from *B. atrox*, and the inhibition by a peptidomimetic compound that could interact with the zinc cofactor of the metalloproteinases present in this venom and in tissue-active metalloproteinases. Some previous observations with plant compounds and analogs of Lapachol show that synthetic naphthoquinones present antineoplasic activity on human malignant cell lines and antileishmanial activity on *Leishmania amazonenses* [69,119]. These activities are related to enzyme inhibition, as shown by data demonstrating the anti-snake venom activity [51]. It is also relevant to mention that Lapachol derivatives possessing indole scaffolds are reported as topoisomerase 1 inhibitors [120].

**Figure 4:**
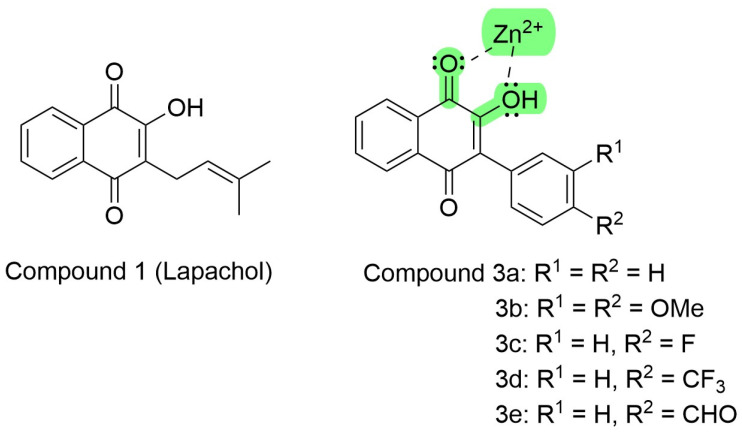
Lapachol chemical structure and its synthetic analogs show the possible pharmacophoric Moyet that can target metalloproteinases (modified from Strauch et al. [54]).

For many years, our group has worked with natural and planned synthetic compounds that could antagonize crotalid venoms or isolated toxins either in vitro or in vivo [5,53,58,62]. Some previous observations, performing different experimental protocols, and searching for natural and synthetic antivenoms, plant compounds, and plants that could work alone as an antivenom or improve antivenom tissue protection, found very positive results [46,56,58,60,62,121]. Our search was initially focused mainly on the antimyotoxic or anti-phospholipase effects of these plants or compounds [29,46,54]. We performed a few experimental observations, testing against *Bothrops* snake venoms with plant extracts or their isolated natural compounds, and searching for antiproteolytic activities either in vivo or in vitro [29,46,53,55]. Among these synthetic-related compounds, we worked with new coumestans and synthetic naphthoquinones. On experimental tests with naphthoquinone for neutralizing venom effects in vitro and in vivo, primarily new synthetic analogs of Lapachol, can be observed in Figure 4 [51,54].

These synthetic compound derivatives from Lapachol could inhibit metalloproteases through the interaction with divalent cations, as ascribed by Preciado et al. [118]. Lapachol derivatives are bidentate ligands, where the electron pair of both the carbonyl and hydroxyl moieties coordinate with the empty orbitals of divalent zinc, as shown in Figure 4.

## 7. Experimental Findings from the Lapachol Analogs on *Bothrops atrox* Effects

Previous investigations, searching for alternative substances that antagonize snake toxins and protect the tissue, led us to find and create prototypes and new planned molecules that could relevantly neutralize snake venoms [51,53]. We found metalloprotease inhibitors devoid of other enzymatic inhibitory effects, such as antimyotoxic or anti-phospholipase A2 activities [51,54]. The results in this investigation allowed us to ascribe that these metalloproteinase and collagenase inhibitors protect the basal lamina in the capillary vessels and prevent hemorrhaging, but do not interfere in the blood coagulation or phospholipase activities of *B. atrox* venom.

**Figure 5:**
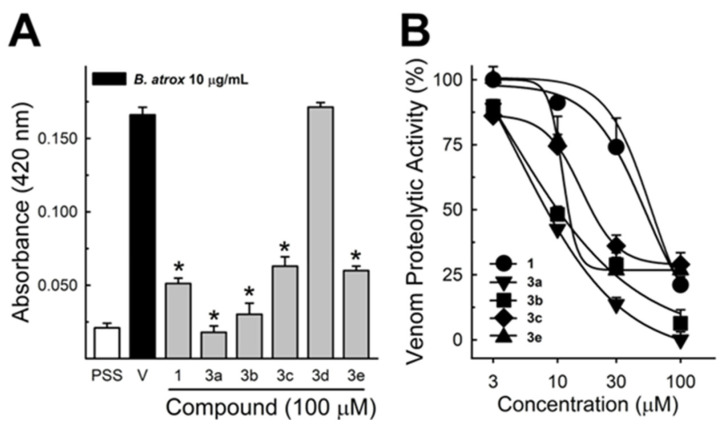
*Bothrops atrox* proteolytic activity inhibition by Lapachol and its synthetic analogs. Proteolytic activity of *B. atrox* venom, and the effect of Lapachol and analogs. (**A**) The inhibition of the venom (10 μg/mL) by Lapachol (**1**) and synthetic analogs (numbers **3a**, **3b**, **3c**, **3d**, and **3e**) at 100 μM (*n* = 5). (**B**) The inhibition of proteolytic activity by Lapachol and compounds in a concentration-dependent way (*n* = 5). One-way ANOVA Dunnett’s post hoc test, * *p* < 0.05, versus *B. atrox* venom (Panels **A**,**B**) (modified from Strauch et al. [54]).

**Figure 6:**
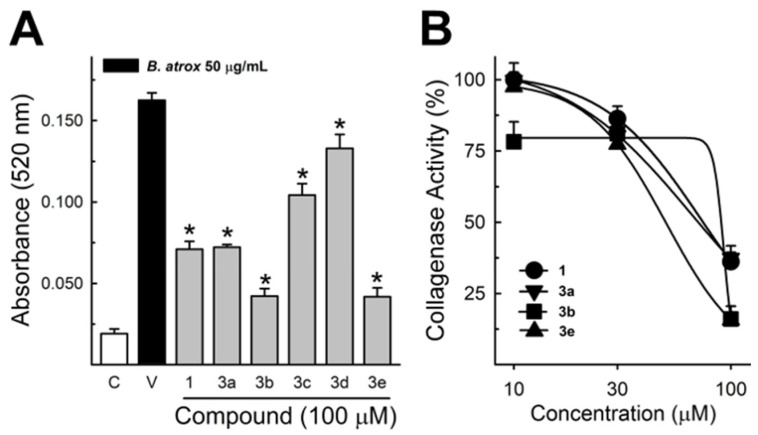
Collagenase activity of *B. atrox* venom, and the effect of Lapachol and analogs. (**A**) The inhibition of *B. atrox* venom (50 μg/mL) by Lapachol (**1**) and analogs (numbers **3a**, **3b**, **3c**, **3d**, and **3e**) at 100 μM (*n* = 5). (**B**) The inhibition of venom collagenase activity by compounds **1**, **3a**, **3b**, and **3e** in a concentration-dependent way (*n* = 5). For all data, one-way ANOVA Dunnett’s post hoc test, * *p* < 0.05, versus *B. atrox* venom (modified from Strauch et al. [54]).

**Figure 7:**
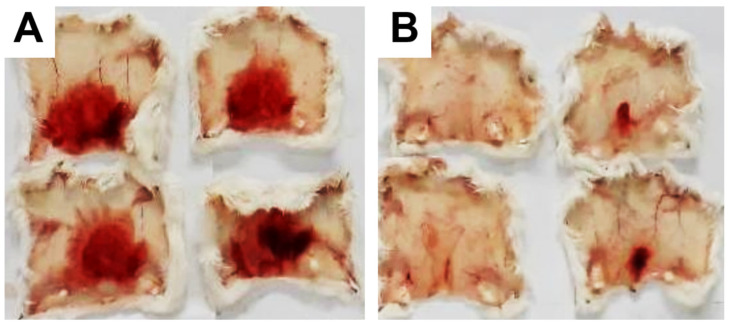
Skin hemorrhage caused by *Bothrops atrox* venom and the effect of Lapachol analogs. Panel (**A**) shows images of the hemorrhagic effect of *Bothrops atrox* venom (1 mg/kg) in mouse skin, demonstrating skin hemorrhage after the intradermic injection; panel (**B**) demonstrates skin hemorrhage after the intradermic injection of venom in the presence of analog **3a** (1 and 3 mg/kg). The quantification data are presented in Figure 8A.

**Figure 8:**
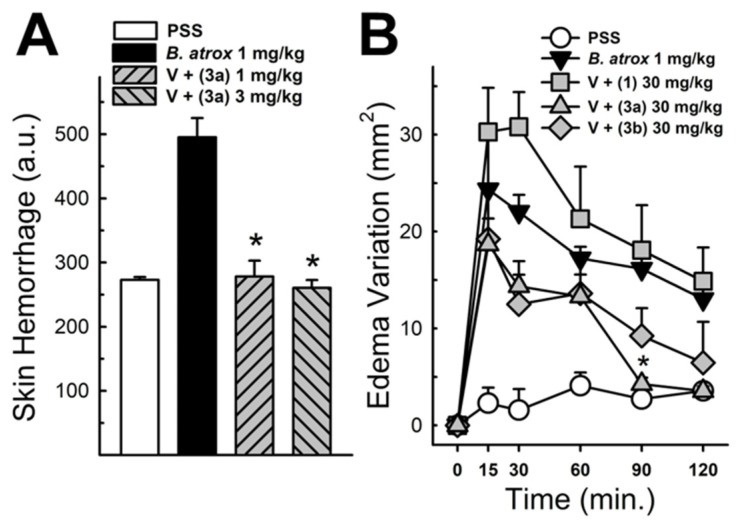
Panel (**A**) shows the hemorrhagic activity of *B. atrox* venoms in mouse skin: the effect of analog **3a**. The data demonstrate skin hemorrhage after the intradermic injection of venom in the presence of analog **3a** (1 and 3 mg/kg). One-way ANOVA Dunnett’s post hoc test, * *p* < 0.05, versus *B. atrox* venom (*n* = 8). Panel (**B**) shows the *B. atrox* edematogenic activity in mice after a tight injection, alone or associated with analog **3a** or Lapachol (30 mg/kg). On panel (**B**) One-way ANOVA Dunnett’s post hoc test, * *p* < 0.05, versus *B. atrox* venom (*n* = 5) (modified from Strauch et al. [54]).

These data were based on previous investigations on Lapachol, and some of the new potential active analogs based on the 2-hydroxy-naphthoquinone scaffold antagonized important activities of *Bothrops* venoms [51] under different experimental protocols in vitro and in vivo. In the present review, we reported on many of our previous investigations on plant extracts, as well as isolated and synthetic compounds, that showed anti-snake venom effects [29,46,51,55,59,60]. We highlighted these results from Strauch et al. [54], mainly showing data antagonizing the proteolytic effects of *B. atrox* venom on the bench, either in vitro or in vivo, on mice. The bioassays performed with venom and compounds included procoagulant, PLA2, collagenase, and proteolytic activities in vitro and in vivo, resulting in venom-induced hemorrhage, edema, and myotoxicity in mice. Lapachol and the synthetic analogs (**3a**, **3b**, **3c**, and **3e**) inhibited, in vitro, the proteolytic and collagenase activities of *B. atrox* venom (Figure 5 and Figure 6, respectively), but did not inhibit the PLA2 activity nor the myotoxic activity. While the analog **3a** inhibited edema, Lapachol did not. In vivo experiments using a crude venom intradermal injection on mouse skin showed skin hemorrhage induced by *Bothrops atrox* (1 mg/kg) and the inhibition of the hemorrhagic effect by the Lapachol analog compound **3a** (Figure 7). The inhibition of the enzymatic activities might help to explain the effects of the analog **3a** in vivo, which decreased and protected mouse skin hemorrhage and edema induced by *B. atrox* venom (Figure 8) [54].

There is no answer in our published data or in our previous investigation explaining why compound **3a** is devoid of a protective effect against myotoxicity. It could be explained the inability to effectively inhibit PLA2 venom activity, while the ability to protect mice from venom hemorrhage could result from the metalloproteinase inhibition on collagenase activity [54]. Our previous data indicated that most of the cell damage promoted by phospholipase toxins was induced by the polycationic properties and the presence of Lysine residues, which the polyanions can neutralize [58,62,114,121]. Previous studies have already established a body of knowledge on metalloproteases, which are involved in many tissues through modulation, remodeling, cytoprotective, or signaling mechanisms, and more molecular approaches are needed to clarify the proteolytic specificity of substrates [96,122,123]. More research is still needed, particularly to examine how molecules interact with each other, to better understand the underlying mechanisms.

## 8. Conclusions

Overall, the synthetic quinone analogs of Lapachol improved the abilities of this compound to neutralize the proteolytic and collagenase activity of *B. atrox* crude venom. Identifying how these synthetic derivatives from Lapachol interact with metalloproteinases should provide insights into these biochemical interactions and their molecular basis. Further research will continue to define the mechanisms for potential therapeutic interventions in snake envenomation or to inhibit endogenous proteases.

## 9. Future Perspectives

Further research will continue to define the mechanisms for potential therapeutic intervention or for endogenous proteases. With advances in the isolation of bioactive substances and the synthesis of new compounds, there is the potential for the development of more effective therapies against venom-induced damage, reducing tissue injuries such as hemorrhage, edema, and necrosis. One of the most important sources and guides of modern therapeutics, the textbook of pharmacology, Goodman and Gilman’s *The Pharmacological Basis of Therapeutics*, in its last edition, describes, in the first chapter, the importance and power of plants and natural compounds in drug development [124]. Further investigations using natural compounds from plants or planned synthetic derivatives to neutralize crude snake venom activities are an alternative way to continue the challenge of developing new antivenoms. Combining venom genomics, new chromatography, proteomic advances, molecular synthesis, and computational modeling approaches, we expect to create safe medicine and improve biomedical research.

## Figures and Tables

**Table 1:** Published articles and terms.

Keywords	PubMed	ScienceDirect	Web of Science
Plants and snakebites	485	1880	393
Plants and snake venom	549	4167	544
Snakebites	6093	4082	3531
*Bothrops* envenomation	2231	1879	643
*Bothrops* and metalloproteinases	460	1182	521
Folk medicine and snakebites	386	584	61
Metalloproteinases	147,270	105,463	52,299
Snake venom metalloproteinases	1550	2360	1022
Lapachol	258	820	550
Lapachol and metalloproteinases	3	24	2
Lapachol and folk medicine	12	50	10
Metalloproteinases inhibitors	48,839	89,688	29,055

## Abbreviations

The following abbreviations are used in this manuscript:

AchEs	Acetylcolinesterases
EP	*Eclipta prostrata*
HAases	Hyaluronidases
HE	Hematoxylin and Eosin
LAAOs	L-amino Oxidases
MMPs	Matrix Metalloproteinases
PDEs	Phosphodiesterases
PLA2	Phospholipase A2
SVMPs	Snake Venom Metalloproteinases
SVSPs	Snake Venom Serine Proteases
WEL	Wedelolactone
WHO	World Health Organization
